# Whakawhiti Kōrero, a Method for the Development of a Cultural Assessment Tool, Te Waka Kuaka, in Māori Traumatic Brain Injury

**DOI:** 10.1155/2015/137402

**Published:** 2015-10-21

**Authors:** Hinemoa Elder, Paula Kersten

**Affiliations:** ^1^Te Whare Wānanga o Awanuiārangi, 13 Domain Drive, Whakatāne 3158, New Zealand; ^2^AUT, Auckland 1010, New Zealand

## Abstract

The importance of tools for the measurement of outcomes and needs in traumatic brain injury is well recognised. The development of tools for these injuries in indigenous communities has been limited despite the well-documented disparity of brain injury. The wairua theory of traumatic brain injury (TBI) in Māori proposes that a culturally defined injury occurs in tandem with the physical injury. A cultural response is therefore indicated. This research investigates a Māori method used in the development of cultural needs assessment tool designed to further examine needs associated with the culturally determined injury and in preparation for formal validation. Whakawhiti kōrero is a method used to develop better statements in the development of the assessment tool. Four wānanga (traditional fora) were held including one with whānau (extended family) with experience of traumatic brain injury. The approach was well received. A final version, Te Waka Kuaka, is now ready for validation. Whakawhiti kōrero is an indigenous method used in the development of cultural needs assessment tool in Māori traumatic brain injury. This method is likely to have wider applicability, such as Mental Health and Addictions Services, to ensure robust process of outcome measure and needs assessment development.

## 1. Introduction

The importance of tools for the measurement of outcomes and needs in traumatic brain injury (TBI) research is well recognized [1]. The development of tools for these injuries in indigenous communities has been limited despite the well-documented disparity of brain injury [2]. The lack of focus on cultural aspects of TBI in international classification systems may also have an influence [3]. Recent analysis and cross-checking of data across several data sets in Aotearoa, New Zealand, including Coroner's Office information, found that young Māori (Māori are the indigenous people of Aotearoa, New Zealand. According to the most recent population census there are approximately 600,000 people identified as Māori, 15% of the general population, or one in seven people. Māori are a steadily growing group, with a 6% increase in population compared to 2006. Population trends show this continuing growth (Statistics New Zealand [4])) are three times more likely to sustain a TBI secondary to violence and that overall incidence of Māori hospitalizations secondary to TBI were also three times that of non-Māori [5]. Māori infants have also been found to have very high rates of subdural haematoma, likely caused by assault [6]. Hence there is a pressing need to develop culturally meaningful assessment measures for Māori. Such measures would also help inform cultural competencies required in the workforce, service development, and the ability to monitor Māori-determined outcomes. Ideally, development of such tools requires processes that can withstand critique from both Te Ao Māori and Western Science.

Despite the well-known Māori belief that he tapu te upoko (the head is sacred), there is a paucity of Rangahau Kaupapa Māori (research by Māori, for Māori, with Māori) and Kaupapa Māori Theory pertaining to TBI [7]. The wairua theory of traumatic brain injury (TBI) in young Māori proposes that a culturally defined injury occurs at the same time as the physical injury [8, 9]. This injury is proposed to occur to wairua, a Māori specific expression of the connectedness between people and the universe, sometimes also translated as the spiritual dimension of being [10]. This means a cultural response is indicated and infers that without response to the cultural injury the whānau recovery will be limited. It is likely that cultural responses would include whānau specific activities that are deemed to address the injury to wairua, these might include deliberate use of Te Reo Māori me ōna tikanga, the Māori language, and protocols such as karakia (prayers) and whakawhanaungatanga (exploring and acknowledging the connections between people). However, there are no tools that assess the extent of this culturally defined injury. To this end, this study aimed to develop a new assessment tool, Te Waka Kuaka.

## 2. Methods

This study used a novel and unique Māori method, whakawhiti kōrero, literally the exchange of ideas and discussion, which predates the concept of cognitive interviewing, as part of mātauranga Māori (Māori knowledge systems) [11]. Whakawhiti kōrero is a term from Te Ao Māori (The Māori world) that expresses active discussion and negotiation. This method has not previously been specifically identified as useful in engaging with Māori participants to develop better statements used in the development of assessment tools. English terms such as “focus groups” [12] or “telephone interviews” [13] have been previously used with Māori participants. However, the premise here is that in using concepts from Te Reo Māori (the Māori language) this approach would promote ease of participation for Māori in the process of developing robust statements in the assessment tool.

Three wānanga (traditional fora) were held in Te Tai Tokerau (Northland), with community health and educational workers. A fourth wānanga with whānau (extended family) with experience of traumatic brain injury was also held to ensure that the statements were acceptable to these whānau, the future end users of such a tool. The initial version of the statements used in the tool came from statements made by participants during the phase of research previously reported [8, 9].

Two wānanga were organized by the author at centres of health work in rural and remote services affiliated tribally and at one meeting of predominantly educational workers. These locations were chosen because of the existing research partnership between these services and the first author's ongoing research programme. The final wānanga was held at a Kaupapa Māori Service provider “Kia ū ki te whānau, whatever it takes,” in order to offer participation to whānau with experience of TBI.

The first two and fourth wānanga were opened with karakia (prayer) and whakawhanaungatanga (introductions that emphasis the kinship linkages of participants). The third wānanga occurred during the lunch break of a hui (meeting) about Māori early childhood education where karakia had opened the day's proceedings. These cultural rituals of encounter are important for many Māori as they ensure culturally safe whakawhiti kōrero. The background rationale of the research was then presented.

The consenting process occurred as part of a presentation about the rangahau by the first author and rationale for this part of the process to each group. The information sheets and consent forms were offered in both Te Reo Māori and English and discussion was invited. The option of having the wānanga filmed was offered. Notes were taken by the author during each wānanga, in particular of the final version of agreed statements from each wānanga.

Data analysis occurred via the author's repeated viewing of wānanga footage and noting commentary regarding the process.

Forty-seven statements were taken from previous research [8, 9]. These statements were in English language with appropriate use of Te Reo (the Māori language). They were presented on individual power point slides at each wānanga. Each statement was read out loud by the first author and comments were invited.

This study received ethical approval from the New Zealand Health and Disability Ethics Committee (14/CEN/17). Consent from the author's whānau was also given by kaumatua (elders) in the author's tribal area of origin.

## 3. Results

A total of thirty-nine people participated in the whakawhiti kōrero in four sites. No participants attended more than one wānanga. Eleven (Kaitaia), six (Te Hapua), sixteen (Waitangi), and six (Henderson) participants consented, respectively. Most (72%, 28) were Māori women, 13% (5) were Māori men, 13% (5) were non-Māori women, and one non-Māori man participated. The first three groups of participants were health and education workers with experience of and interest in working with Māori whānau. The final group were whānau who had direct experience of TBI.

Many of the original 47 statements, 23 (49%), were changed or removed during at least one wānanga. One new statement was added by the Waitangi wānanga “*Waiata is healing for those who do not understand Te Reo,*” the comments being that waiata, song, is such a common and important cultural practice that needed to be included.

Two statements were rejected by the participants, firstly, “*in hospital they treat the sickness not the wairua Māori that needs to be treated.*” This concept was put “on hold” by the first wānanga who found it “difficult to put into practice” and did not feel it would be easy to respond to. Subsequent wānanga participants concurred so the statement was removed from the final version of the assessment tool. In addition, the statement, “*time when whānau gather together builds healing,*” was thought to be almost exactly the same as “*whānau unity and strength builds healing*” and was therefore excluded.

One striking response was the participants dislike of the words “clinician” and “professional” with a preference for the terms “health worker” and “kaimahi” (worker). The participants discussed that they found words such as “clinician” distanced whānau from relating to the subject and to engaging in the process. They felt the concept of health worker was more inclusive and ensured inclusion of “Whānau Ora workers (Whānau Ora is a specific type of contract to workers who are cross-disciplinary and who work to facilitate whānau rangatiratanga (self-determination) Turia [14].)”. In addition, words such as “attended to” were found to be clumsy and were adapted. The reworking of statements commonly brought meaning for whānau to the fore. Collective ownership was taken around the concept of whānau, with the phrase “our whānau” being adopted in one statement.

The initial statement:

* “the first thing that needs to happen is for wairua to be attended to,” *

was modified by the Waitangi wānanga to

*“starting the process of wairua healing is the first thing that needs to occur for our whānau” *

and again by the Henderson wānanga to

* “starting the wairua healing is the first thing that needs to happen for our whānau.”*

Another reason for modification of statements was to refine the extent to which the statement reflected the majority of a concept or ameliorated that. Shifting from the concept of “most” to “some” and then to removing that modifier and including the concept “could” resulted in a statement that participants felt would be easier to respond to.

The initial statement:

*“clinicians do not take enough time explaining what is going on to whānau,”*

was modified by the Kaitaia wānanga to

*“most health workers do not take enough time explaining what is going on for whānau.”*

This was further modified at the Te Hapua wānanga to

*“some health workers do not take enough time explaining what is going on with whānau.”*

This was again refined by Waitangi to become

*“health workers could take more time to help the whānau to understand what is going on.”*

As the wānanga progressed the degree to which concepts of health workers, taking time, and meeting needs of whānau were woven together evolved to focus the statement more on the sense of responsibility of the kaimahi to bring the concept of flexibility to their work schedule in order to meet whānau needs.

The initial statement:

*“clinicians expect whānau to take up as little of their time as possible,”*

was modified in Kaitaia to

*“most health workers expect whānau to take up as little of their time as possible.”*

This was further adjusted in Te Hapua to

*“some health workers schedules don't match the needs of whānau.”*

And finally Waitangi wānanga refined the statement to

*“it is important that kaimahi (workers) are flexible in their schedules of work.”*

Some statements began framed in a negative sense. Most were reframed in a positive way.

The initial statement:

*“I get upset if time is not taken for wairua,”*

was modified in Waitangi to

*“I feel uplifted when time is taken for wairua.”*

The group with whānau experience of TBI tended to strengthen statements regarding impact on whānau and with a positive reframe such as in this example.

The initial statement:

*“professionals are not trained in the culture of the whānau,”*

was modified in Waitangi to

*“health workers do not always relate to the culture of the whānau”*

and was further modified in Henderson to

*“when health workers relate to the culture of the whānau, outcomes are improved.”*

Half of the statements remained unchanged. These were most strongly clustered in the areas of “wairua practices.” For example,

*“It doesn't matter if you can't understand Te Reo (the Māori language), the effect remains strong”, Karakia strengthens wairua, the closeness of whānau strengthens wairua, mirimiri (massage) strengthens wairua, use of Te Reo Māori means wairua is being strengthened.”*

A final version of the assessment tool called Te Waka Kuaka is now ready for validation procedures (see the Appendix). The name Te Waka Kuaka was chosen as it describes a flock of kuaka (godwits). The kuaka holds special significance for the iwi (tribal group) of the first author. Te Waka Kuaka supports whānau to organize their thoughts and needs which then enables them to navigate their healing journey, much as the flock of kuaka organizes themselves for their long migrations around the globe. This name was welcomed by kaumatua (elders).

## 4. Discussion

It was notable that participation was not declined by any of the potential participants. It is possible that this is related to at least two aspects. Because of the longstanding research partnership between the first author and these groups there was a considerable amount of historical trust accrued. This may have assisted in enabling participants to more fully engage. In addition, the use of Māori protocols of encounter such as karakia and whakawhanaungatanga assisted in ensuring that all participants and the author could locate each other within the bonds of their collective ancestral ties. Without this history and use of cultural lore it is possible that the participants may have been less receptive to the whakawhiti kōrero.

The whakawhiti kōrero approach was well received by participants. They seemed to require little prompting as to their ideas about how the statements might best flow and be understood and responded to in the future. Key considerations at the Kaitaia meeting were that the participants did not feel the words “clinician” and “professional” were appropriate and might be off-putting for future respondents. The Waitangi meeting strongly advocated for turning negative statements into positive ones. However, there remain four statements with a negative element (11, 25, 39, and 45): “being inside buildings like hospitals does not help me,” “whānau switch off when they hear the word “clinical,”” “separating the whānau from the patient can damage healing,” and “it does not matter if you cannot understand Te Reo, the effect remains strong.” It is possible that the majority of statements formed in an affirmative manner reflect a strong preference for a nondeficit approach which resonated for the participants. These preferences are also articulated by Kaupapa Māori Theory and praxis scholars [15, 16]. Across all wānanga the participants discussed their ideas amongst themselves and came to shared agreement as to changes and to statements they did not wish to change.

While not specifically critiqued, the power of naming the process from within Te Ao Māori, the Māori world view, as whakawhiti kōrero seemed to contribute to an overall ease of participation and engagement in this study. The positive impact of using Māori ways of being from daily life as research methods rather than treating these as “other” in the research space has previously been described [17].

At each wānanga kaimahi from areas of health such as chronic care nurses and Whānau Ora workers commented that they would like to use the tool in their work, unrelated to TBI. The view expressed was that the tool would be useful to build rapport and to obtain a clear, shared profile of the cultural needs of whānau, whatever their index health concerns. The first author expressed caution while the research process was occurring and also suggested that future projects could be developed to investigate this possible use.

The development of robust assessment tools commonly involves the process of cognitive interviewing [18]. There is no universally accepted definition of this method. However, it involves practices that present draft questionnaire statements to participants, one to one interviews being the described modality, and using various techniques such as asking participants to think out loud, in order to help improve the statements such that they deliver the information needed. While some may understand whakawhiti kōrero as a modification of cognitive interviewing it is best understood as a related but distinct method that comes from Māori mātauranga (Māori knowledge systems). Building a wider vocabulary of Māori research methods is a critical issue which has attracted considerable attention [16]. In particular, the need to make visible the “normal” aspects of Māori knowledge and practice has been highlighted as a site of rich and important methodological resource [17]. The use of a culturally determined meeting context such as wānanga demonstrated that group processes can be successfully used to refine assessment tool statements in preparation for validation.

This study is limited by its location in the north of Aotearoa, New Zealand. It is possible that the response to statements and the participant engagement would be varied in other areas. Given the original research that identified the forty-seven initial statements coming from marae wānanga (fora in traditional meeting houses) in nine urban, rural, and remote locations around Aotearoa, New Zealand, this reinforces the wide applicability of the results.

## 5. Conclusion

Whakawhiti kōrero is an indigenous concept that brings a practice from Māori daily life to serve as a method in Rangahau Kaupapa Māori. This method has been utilised in the development of cultural needs assessment tool in Māori traumatic brain injury in order to refine the tool based on robust theory building. This method may have wider applicability in other fields where outcome measures and needs assessments for Māori are needed such as mental health and addiction services and in assessment treatment of neurodegenerative disorders and others. This approach could itself be examined in more detail as to how it is received by participants and the features that this approach contributes to participation by Māori. This method adds to the suite of Rangahau Kaupapa Māori processes that map out a robust process of outcome measure and needs assessment development. A final version of the assessment tool, Te Waka Kuaka, is now ready for validation procedures.

## Appendix

*Te Waka Kuaka. Cultural Needs of Whānau with Possible Brain Injury*

*Date*

*Page 1 ID*……

Relationship of participant to patient
Main iwi of the participant
Age of participant
Gender of participant
Ethnicity of health worker
Profession of health worker
Age of health worker
Gender of health worker
Location area name
Mental health issues/addiction/birth hypoxia (circle)
Possible TBI (circle) date (s)
Confirmed TBI (circle) date (s)
  □ Mild (Mild)
  □ Moderate (Mod)
  □ Severe (Sev)
  □ Unknown (circle) (Unk)
1 = strongly disagree
2 = disagree
3 = agree
4 = strongly agree

* Wā (Time)*

1. Starting the process of wairua healing is the first thing that needs to happen for our whānau
  □ 1
  □ 2
  □ 3
  □ 4
2. The journey of wairua healing for whānau is enhanced with time
  □ 1
  □ 2
  □ 3
  □ 4
3. Whakawhanaungatanga at the beginning sets the scene for the journey
  □ 1
  □ 2
  □ 3
  □ 4
4. Health workers could take more time to help the whānau understand what is going on
  □ 1
  □ 2
  □ 3
  □ 4
5. It is important that kaimahi are flexible in their schedules of work
  □ 1
  □ 2
  □ 3
  □ 4
6. I get uplifted when time is taken for wairua
  □ 1
  □ 2
  □ 3
  □ 4
7. Time needs to be taken to consider other trauma within whakapapa
  □ 1
  □ 2
  □ 3
  □ 4
8. Whanaungatanga time builds, to keep hope and dreams alive
  □ 1
  □ 2
  □ 3
  □ 4
9. Whānau unity and strength builds healing
  □ 1
  □ 2
  □ 3
  □ 4
10. Comments – – – – – – – – – – – – – – – – – – – – – – – – – – – – – – – – – – – – – – – – – – – – – – – – – –

*Wāhi (Place)*

(10) The use of pepeha within treatment would support the healing
  □ 1
  □ 2
  □ 3
  □ 4
(11) Being inside buildings like hospitals does not help me
  □ 1
  □ 2
  □ 3
  □ 4
(12) It makes me feel better when we can go to the marae
  □ 1
  □ 2
  □ 3
  □ 4
(13) Whakaairo (carvings) teach important lessons that help with healing
  □ 1
  □ 2
  □ 3
  □ 4
(14) Tukutuku (lattice-work) panels have important lessons for healing
  □ 1
  □ 2
  □ 3
  □ 4
(15) The powhiri process ensures the wairua is settled for open discussion
  □ 1
  □ 2
  □ 3
  □ 4
(16) Gathering, preparing, and eating food from home is an important part of healing
  □ 1
  □ 2
  □ 3
  □ 4
(17) Whānau from home are an essential link with home
  □ 1
  □ 2
  □ 3
  □ 4
(18) Māori may feel the need to come home to heal
  □ 1
  □ 2
  □ 3
  □ 4
(19) Being on the marae is a good place to start to feel strong again
  □ 1
  □ 2
  □ 3
  □ 4
Comments – – – – – – – – – – – – – – – – – – – – – – – – – – – – – – – – – – – – – – – – – – – – – – – – – –

* Te Waka Kuaka Measure for Whānau with Possible TBI*

*Page 2 ID*……

*Tangata (People)*

(20) When the whānau are involved the healing outcome is better
  □ 1
  □ 2
  □ 3
  □ 4
(21) Whānau have to go through their own healing process
  □ 1
  □ 2
  □ 3
  □ 4
(22) Within whānau there are a lot of resources
  □ 1
  □ 2
  □ 3
  □ 4
(23) Within the whānau is the rongoā
  □ 1
  □ 2
  □ 3
  □ 4
(24) Whānau fear judgment by health workers
  □ 1
  □ 2
  □ 3
  □ 4
(25) Whānau switch off when they hear the word “clinical”
  □ 1
  □ 2
  □ 3
  □ 4
(26) Māori have a different point of view from Pākehā
  □ 1
  □ 2
  □ 3
  □ 4
(27) Māori cultural needs are different from Pākehā
  □ 1
  □ 2
  □ 3
  □ 4
(28) When health workers relate to the culture of the whānau outcomes are improved
  □ 1
  □ 2
  □ 3
  □ 4
(29) When health workers support whānau to address wairua outcomes are improved
  □ 1
  □ 2
  □ 3
  □ 4
(30) I call on the strengths of my tūpuna to cope
  □ 1
  □ 2
  □ 3
  □ 4
(31) Trauma to one is trauma to all
  □ 1
  □ 2
  □ 3
  □ 4
(32) Trauma to one is trauma to the whakapapa
  □ 1
  □ 2
  □ 3
  □ 4
(33) Being whānau means you do not have to know everything yourself
  □ 1
  □ 2
  □ 3
  □ 4
(34) Being whānau means we can use our collective strengths
  □ 1
  □ 2
  □ 3
  □ 4
Comments – – – – – – – – – – – – – – – – – – – – – – – – – – – – – – – – – – – – – – – – – – – – – – – – – –

*Wairua Practices*

(35) Practices that strengthen wairua are as important as clinical interventions
  □ 1
  □ 2
  □ 3
  □ 4
(36) Karakia strengthens wairua
  □ 1
  □ 2
  □ 3
  □ 4
(37) The presence of kaumatua strengthens wairua
  □ 1
  □ 2
  □ 3
  □ 4
(38) The closeness of the whānau strengthens wairua
  □ 1
  □ 2
  □ 3
  □ 4
(39) Separating whānau from the patient can damage healing
  □ 1
  □ 2
  □ 3
  □ 4
(40) Te Reo Māori me ōna tikanga is important in maximizing healing of wairua
  □ 1
  □ 2
  □ 3
  □ 4
(41) Oriori (chants) can be powerful healing tools
  □ 1
  □ 2
  □ 3
  □ 4
(42) Mirimiri (type of massage) can be a powerful healing tool
  □ 1
  □ 2
  □ 3
  □ 4
(43) Romiromi (type of massage) can be a powerful healing tool
  □ 1
  □ 2
  □ 3
  □ 4
(44) Waiata is healing for those who do not understand Te Reo
  □ 1
  □ 2
  □ 3
  □ 4
(45) It does not matter if you cannot understand Te Reo, the effect remains strong
  □ 1
  □ 2
  □ 3
  □ 4
(46) Use of Te Reo Māori means wairua is being strengthened
  □ 1
  □ 2
  □ 3
  □ 4
Comments – – – – – – – – – – – – – – – – – – – – – – – – – – – – – – – – – – – – – – – – – – – – – – – – – –
                               copyright Dr H. Elder
